# Nursing home nurses’ and community-dwelling older adults’ reported knowledge, attitudes, and behavior toward antibiotic use

**DOI:** 10.1186/s12912-017-0203-9

**Published:** 2017-03-11

**Authors:** Christine E. Kistler, Anna Beeber, Sylvia Becker-Dreps, Kimberly Ward, Megan Meade, Brittany Ross, Philip D. Sloane

**Affiliations:** 1 Cecil G. Sheps Center for Health Services Research, Department of Family Medicine, University of North Carolina, Chapel Hill, NC USA; 2 Cecil G. Sheps Center for Health Services Research, School of Nursing, University of North Carolina, Chapel Hill, NC USA; 3 Department of Family Medicine, University of North Carolina, Chapel Hill, NC USA; 4 Cecil G. Sheps Center for Health Services Research, University of North Carolina, Chapel Hill, NC USA; 5 School of Medicine, University of North Carolina, Chapel Hill, NC USA

**Keywords:** Infection management, Older adults, Primary care, Nursing homes

## Abstract

**Background:**

Antibiotic overuse causes antibiotic resistance, one of the most important threats to human health. Older adults, particularly those in nursing homes, often receive antibiotics when they are not indicated.

**Methods:**

To understand knowledge, attitudes, and behaviors of nursing home (NH) nurses and community-dwelling older adults towards antibiotic use, especially in clinical situations consistent with antibiotic overuse, we conducted a mixed-method survey in two NHs and one Family Medicine clinic in North Carolina, among English-speaking nurses and community-dwelling, cognitively intact adults aged 65 years or older. Based on the Knowledge-Attitude-Practice model, the survey assessed knowledge, attitudes, and behavior towards antibiotic use, including three vignettes designed to elicit possible antibiotic overuse: asymptomatic bacteriuria (ASB), a viral upper respiratory illness (URI), and a wound from a fall.

**Results:**

Of 31 NH nurses and 66 community-dwelling older adults, 70% reported knowledge of the dangers of taking antibiotics. Nurses more often reported evidence-based attitudes towards antibiotics than older adults, except 39% agreed with the statement “by the time I am sick enough to go to the doctor with a cold, I expect an antibiotic”, while only 28% of older adults agreed with it. A majority of nurses did not see the need for antibiotics in any of the three vignettes: 77% for the ASB vignette, 87% for the URI vignette, and 97% for the wound vignette. Among older adults, 50% did not perceive a need for antibiotics in the ASB vignette, 58% in the URI vignette, and 74% in the wound vignette.

**Conclusions:**

While a substantial minority had no knowledge of the dangers of antibiotic use, non-evidence-based attitudes towards antibiotics, and behaviors indicating inappropriate management of suspected infections, most NH nurses and community-dwelling older adults know the harms of antibiotic use and demonstrate evidence-based attitudes and behaviors. However, more work is needed to improve the knowledge, attitudes and behaviors that may contribute to antibiotic overuse.

## Background

Overuse of antibiotics causes widespread and damaging consequences to human health including the development of antibiotic-resistant bacteria [1]. In the United States (U.S.) alone, mortality from antibiotic-resistant bacteria may be as high as 100,000 persons annually [2]. In addition to increasing mortality, antibiotic resistance magnifies the costs of treatment, prolongs the antibiotic course, and causes adverse events and harmful sequelae, such as the epidemic of *clostridium difficile* [3, 4]. It is particularly important to study use of antibiotics among older adults because, in contrast to other age groups, rates of antibiotic use in this population are high and growing [5]. At any given time, 10.8% of nursing home (NH) residents are receiving systemic antibiotics (i.e., given by mouth or injection) [6]. This widespread use encourages the development of antibiotic resistance [7]. One key cause is asymptomatic bacteriuria (ASB)– a common condition in older persons that is frequently mistaken for infection [8], and which contributes to the finding that up to 70% of prescriptions in NHs do not meet clinical guidelines for appropriate prescribing [9, 10].

These factors explain the current emphasis on “antibiotic stewardship,” i.e., the conservation of antibiotics wherein they are only used when needed, which results in lower overall antibiotic use, thus reducing the expansion of resistant bacteria [11]. However, efforts to curb antibiotic use in older adults have primarily targeted physicians and have had limited impact [12, 13]. A possible reason for this lack of antibiotic stewardship in NHs may be a lack of involvement of nursing staff and patient/family in prescribing decisions. In one study, for example, only 29% of NH residents with unstable vital signs were examined by a provider on-site or prior to transfer to a hospital [14]. Nurses may be the only professional to assess an ill NH resident; as such, nurses’ attitudes may be critical to improving infection management and antibiotic overuse [15]. Therefore, understanding nurses’ attitudes towards antibiotic use in their own care may provide insights into their practice behaviors.

Older adults may also influence prescribing by requesting antibiotics for non-evidence-based reasons [16], such as the request for antibiotics to treat upper respiratory infections (URIs), for which they are not indicated. A study conducted in 1998 showed that one-third of primary care patients living in the community expected antibiotics for a URI [17]. Unfortunately, replication of this study in the NH setting is not possible, due to high rates of dementia [18], and communication difficulties [19]. However, a survey of community-dwelling older adults’ knowledge, attitudes, and behaviors may represent their likely views if admitted to residential care in the future. Though community-dwelling older adults are healthier than their NH counterparts and their knowledge, attitudes, and/or behavior may change, given time constraints, we thought this would be a good first step to examine underlying beliefs and attitudes.

Using the Knowledge-Attitude-Practice model [20] as our framework for understanding antibiotic use, we sought to address the current gap in our understanding of antibiotic use in NHs by examining NH nurses’ and community-dwelling older adults’ knowledge, attitudes, and behaviors towards suspected infections. Additionally, we sought to examine if significant differences exist between nurses and older adults, implying that intervention strategies would need to be tailored to the target population and that one intervention is unlikely to work for all key targets.

## Methods

### Study design

We conducted a mixed-method study of convenience samples of NH nurses and community-dwelling older adults. The nurse and older adult parts of the study were conducted in parallel and mirrored one another in aim and content. Nurses were recruited from two NHs, and community-dwelling older adults were recruited from a university-based Family Medicine clinic; all of which were located in North Carolina. We interviewed all available and interested nurses during two 24-hour periods, one weekend and one weekday. In the Family Medicine clinic we collected appointment times of adults who were 65 years or older and subsequently approached each patient to assess interest. If interested, they were then screened for eligibility, and, if eligible, invited to provide consent to participate. All interviews were conducted in a quiet, private location, except for one older adult who requested to participate over the telephone. The one-time interview contained questions to quantify their knowledge, attitudes, and behavior towards antibiotics together with open-ended probes on their rationales for how they responded to the three clinical vignettes. Most items were the same in both surveys with slightly altered wording for context. Unique participant demographics included years of employment for nurses and marital status and self-reported health for the older adults. Responses to closed-ended questions were recorded on a hard-copy survey during the interview. Open-ended responses were recorded on a tape recorder during the interview and typed into the database, verbatim. Nurses received $25 gift cards for their time, and older adults received a $10 gift card. Study methods were approved by the Institutional Review Board of the University of North Carolina at Chapel Hill.

### Participants

Nurses were eligible for the study if they were fluent English speakers, worked more than 20 h per week, and worked more than four consecutive hours per shift during one of two 24-hour periods (one week day and one weekend). Older adults were eligible for the study if they were current patients at the Family Medicine clinic, fluent in English, aged 65 years or older, and cognitively intact, based on a score of four or more on a six-item screening questionnaire [21].

### Measures

To assess participants’ knowledge and personal attitudes towards antibiotic use, we used four previously developed questions [22]. First, we asked participants to respond to the knowledge question, “Are you aware of any health dangers to yourself or other people associated with taking antibiotics?” We considered a “no” response to be a lack of knowledge about the risk of antibiotic use. Personal attitudes towards antibiotic use were measured with three 5-point Likert-statements, ranging from “strongly disagree” to “strongly agree”, about antibiotic use when they had a cold [22].

To assess behavior, we developed and included clinical vignettes about the three most common conditions seen in older adults for which antibiotics are often not indicated but commonly prescribed: ASB, URIs, and wounds [23]. The clinical vignettes can be found in Table 1. Vignettes have been used to assess both knowledge and behaviors around antibiotic prescribing [24]. Clinical vignettes for physicians appear to be a valid and comprehensive way to assess actual prescribing behaviors [25]. Vignettes have been used to assess both adult and nurse behaviors [26–28]. Older adults were asked, “What do you think you would do in a situation like this?” and nurses were asked, “Though the doctor would make the final decision, do you think < patient name/you > need(s) an antibiotic?”

**Table 1:** Sample clinical vignettes: conditions for which antibiotics are generally not recommended

Condition	Respondent group	
	Nursing home nurses	Community-dwelling older adults
Asymptomatic bacteriuria vignettes	Mrs. Jones is an 83 year old woman with left-sided weakness from a stroke. Your medical assistant informs you that “she has really smelly urine.” At her baseline, she is incontinent of bowel and bladder and non-ambulatory. Her functional status is unchanged, her temperature is 97.8 F, her other vital signs are normal, and she appears to otherwise be in her usual state of health except for her smelly urine.	Mrs. Jones is an 83 year old woman who thinks her urine is more smelly lately. At her baseline, she has occasional urinary incontinence, uses a wheelchair to get around after she had a stroke, and has a hard time getting to the bathroom because it. She doesn’t have a fever, and she appears to otherwise be in her usual state of health except for her smelly urine.
Upper respiratory infection vignettes	Mrs. Smith is a 91 year old woman with moderate dementia and heart failure. You notice she has a cough, runny nose, and low grade fever, but otherwise seems well. Her appetite is still good, her last recorded temperature was 97.4, her other vital signs are normal.	Mrs. Smith is a 91 year old woman with a little memory trouble and heart failure. She starts to have a cough, runny nose, and low grade fever, after a visit with her family, but otherwise seems well with no trouble breathing. She is eating and drinking fine and hasn’t had a high fever.
Wound vignettes	Mr. Jones is an 82 year old with long-standing diabetes. He had a witnessed fall while standing and scraped his forearm catching himself from his fall. He is otherwise uninjured and did not hit his head. He is able to ambulate immediately thereafter and the wound is a superficial skin tear without deep tissue injury.	Mr. Jones is an 82 year old with diabetes for a long time. He fell when getting up from breakfast this morning and scraped his forearm catching himself from his fall. He is otherwise uninjured and did not hit his head. He can walk without any trouble after the fall. The scrape is not very deep, just a scratch barely through the skin, bleeding a little bit.

### Analysis

We used descriptive statistics to describe participant characteristics. Comparisons between the two populations were made using Fisher’s exact tests, given the small sample size. Quantitative analyses were conducted using STATA 11.0.

To assess behavior, two researchers (CK and BR for the nursing survey and CK and MM for the older adult survey) independently read the transcripts and coded the open-ended responses as to whether a participant felt antibiotics were indicated (coded either as yes-indicated, uncertain if indicated, or no-not indicated). Coding discrepancies were discussed among the group and resolved by consensus. We then analyzed the open-ended responses for recurrent themes. Two researchers (CK and BR for the nursing survey and CK and MM for the older adult survey) independently read the transcripts and identified themes in the responses for each group (nurses and older adults) separately. The themes were refined and revised based on further analysis by the team.

## Results

Thirty-one of 32 eligible nursing staff (97%) and 66 of 86 eligible older adults (77%) participated in the interview. The nurses were predominantly middle-aged women, about one-half of whom had a Bachelor's of Nursing Degree or higher level of education. The older adults’ mean age was 72 years old and the majority were white and female (Table 2).

**Table 2:** Participant characteristics, n (%)

Participant characteristics	Nurses (*n* = 31)	Older adults (*n* = 66)
Age, mean ± SD	43 ± 13	72 ± 6
Female	27 (87%)	41 (62%)
Race		
White or Caucasian	11 (35%)	51 (77%)
Black or African-American	16 (52%)	13 (20%)
Asian-American	4 (13%)	1 (2%)
Other	0	1 (2%)
Educational status		
Some high school or less	0	12 (18%)
12th Grade Graduation or GED	0	15 (23%)
Some College or Associate’s Degree, including LPN	15 (48%)	13 (20%)
College Degree, including Bachelor’s of Nursing	15 (48%)	10 (15%)
Masters, PhD, JD, or MD, including Masters of Nursing	1 (3%)	16 (24%)
Antibiotics in the past 6 months	4 (13%)	20 (30%)
Marital status (community sample only)		
Married/Partnered	--	35 (53%)
Widowed	--	19 (29%)
Divorced	--	10 (15%)
Never married	--	2 (3%)
Self-reported health status (community sample only)		
Excellent	--	8 (12%)
Very good	--	16 (24%)
Good	--	28 (42%)
Fair	--	13 (19%)
Poor	--	1 (2%)
Years nursing experience (nurse sample only)		
Less than 2 years	5 (16%)	--
2–5 years	5 (16%)	--
6–10 years	4 (13%)	--
11–15 years	1 (3%)	--
16–20 years	4 (13%)	--
Greater than 20 years	12 (39%)	--
Years worked at site (nurse sample only)		
Less than a year	13 (42%)	--
1–2 years	8 (26%)	--
3–5 years	7 (23%)	--
6–10 years	2 (6%)	--
11–15 years	0 (0%)	--
Greater than 15 years	1 (3%)	--

### Knowledge and attitudes toward suspected infections

About 70% of both NH nurses and community-dwelling older adults reported knowledge of the dangers of taking antibiotics (Table 3). Nursing attitudes towards antibiotics differed significantly from community-dwelling older adults. While virtually all nurses disagreed or strongly disagreed that antibiotics can prevent worsening symptoms, only 71% of older adults did (*p* < 0.01). Conversely, more nurses than older adults agreed or strongly agreed that if they are sick enough to see a doctor for a cold, they expect an antibiotic to be prescribed (*p* = 0.07). However, nurses and older adults did not significantly differ on the proprotion who disagreed or strongly disagreed with the statement, “When I get a cold, antibiotics help me to get better more quickly” (*p* = 0.70).

**Table 3:** Responses to nursing home nurses and community-dwelling older persons to questions about the management of suspected infections, n (%)

Quantitative responses	Nurses (*n* = 31)	Older adults (*n* = 66)	P-value*
Knowledge- Are you aware of any health dangers to yourself or other people associated with taking antibiotics?^a^			
Aware	22 (71%)	45 (69%)	1.0
Attitude- When I have a cold, I should take antibiotics to prevent getting a more serious illness.			
Strongly disagree or disagree	29 (94%)	51 (77%)	0.003
Neutral	0	3 (5%)	
Strongly agree or agree	2 (6%)	12 (18%)	
Attitude- When I get a cold, antibiotics help me to get better more quickly.			
Strongly disagree or disagree	25 (81%)	46 (70%)	0.698
Neutral	2 (6%)	4 (6%)	
Strongly agree or agree	4 (13%)	16 (24%)	
Attitude- By the time I am sick enough to talk to or visit a doctor because of a cold, I usually expect a prescription for antibiotics^b^			
Strongly disagree or disagree	12 (39%)	39 (59%)	0.069
Neutral	6 (19%)	9 (14%)	
Strongly agree or agree	12 (39%)	18 (28%)	
Behaviors- Did not perceive need for an antibiotic in the:			
Asymptomatic bacteriuria vignette	24 (77%)	33 (50%)	0.008
Viral upper respiratory vignette	27 (87%)	38 (58%)	<0.001
Wound vignette^a^	30 (97%)	48 (74%)	0.016

*Statistical method used = Fisher’s Exact

^a^ *n* = 30 for the nurses survey

^b^ *n* = 65 for the older adults survey

### Behavior toward suspected infections

In total, 32% of nurses and 29% of older adults mentioned the need for antibiotics in any of the three clinical vignettes, however more nurses rejected the need for antibiotics more often than older participants in response to all three vignettes (Table 3).

### Qualitative themes

The qualitative analysis identified six themes across all 3 vignettes among participants (Table 3). Four themes were found in both nurses and older adults:

- Further observation: Both nurses and older adults mentioned that the patients needed close observation. A nurse reported, “*I’d keep monitoring her.”* An older adult said, “*I’d just keep an eye on it for now.”*
- Further work-up: Both groups discussed the need for further testing to rule out an infection. In response to the URI vignette, a nurse stated that, *“I’d see if we can get a chest X-RAY.”* In response to the ASB vignette, an older adult mentioned that, *“She needs to get a urinalysis.”*
- Medical provider’s evaluation: Both groups also reported that contacting and interfacing with a medical provider was important. For example, a nurse said, *“I’d notify the doctor, he may want to do something.”* Similarly a common older adult response was, *“I’d want to see the doctor and get it checked out.”*
- Non-pharmacologic management: Both groups noted the need for other interventions besides antibiotics. In response to the wound vignette, a nurse said, *“I’d clean it and dress it.”* In response to the ASB vignette, an older adult said that, *“I’d try to drink lots of water.”*

Two other themes also emerged uniquely from each of our two study populations.

- Follow a protocol: Unique to nurses, several mentioned following wound or fall protocols including the importance of documentation. For the wound vignette, one nurse stated, *“I’d follow the wound care protocol,”* and another stated, *“I’d fill out an incident report on the fall, and then notify the doctor and family.”* For the ASB vignette, another stated that, *“If it’s a new change, I have to fill out a condition change report.”*
- Uncertainty: Unique to older adults, a common theme was general uncertainty about a condition. When asked if they thought each of the vignettes needed an antibiotic, many responded with phrases like, *“I have no way of knowing”*, *“That’s something I couldn’t say”*, and *“I don’t know, I’m not sure.”*

## Discussion

We found that the majority of NH nurses and community-dwelling older adults had knowledge about the dangers of antibiotics, though their attitudes toward the use of antibiotics differed. A sizeable minority of nurses and older adults reported that antibiotics were warranted in hypothetical situations where they are unlikely to be beneficial. For older adults, open-ended responses revealed uncertainty about the need for antibiotics. For nurses, response yielded potential targets for future interventions.

The number of nurses with knowledge of the dangers of antibiotics was high. While no specific survey of nurse knowledge or attitudes towards suspected infections exists for comparison, most nurses disagreed with the use of antibiotics if they had a common cold, though 39% felt that if they were sick enough to see a physician they usually expected an antibiotic. These findings suggest that while most nurses appear to understand the dangers of antibiotics, a minority may have personal attitudes that may increase overuse of antibiotics for themselves. This perceived need for an antibiotic may be due to the fact that nurses believe they wouldn’t see a medical provider unless they had symptoms severe enough to warrant an antibiotic, because only four nurses felt an antibiotic was needed for the viral URI vignette. Reassuringly, only a minority of nurses reported the need for antibiotics in our three clinical vignettes, which may represent discordance between their personal attitudes and practice behavior in need of future research. A survey of 192 U.S. nurses interested in geriatric nursing found that 80% felt malodorous urine was sufficient to start work-up for a UTI [29], behavior unsupported by the Loeb criteria [30]. In contrast, our results showed that 77% of nurses did not believe a malodorous urine warranted antibiotics. Similar to the findings of a qualitative study in Australia of 40 nurses which found guidelines to be an important work-flow factor in nursing infection management [16], nurses identified that following a protocol is important for managing infections. They reported a medical provider’s input to be important, as also found in the Australian study. If nurses endorse the use of protocols for falls, then protocols for other nursing home issues such as a suspected infection may provide high-yield strategies for improving behavior [31]. This concept is reinforced by a recent clinical protocol developed in 8 NHs in Canada and 3 in Iowa to help NH nurses manage suspected UTIs that found general nursing support for its use [32].

A majority of older adults also reported knowledge of the dangers of antibiotics. Prior work a decade ago found that 58% of adult patients were not aware of any dangers of antibiotic use [22], compared to only 29% in our sample. Attitudes varied by context but about 25% of older adults had non-evidence-based attitudes towards antibiotics. In general, patients expect quicker symptom resolution than is warranted by the evidence. For example, a recent systematic review found that adults in the outpatient setting expected a cough from a viral URI to last about 8 days, though actual duration is closer to 18 days [33]. While most of the older adults in the current study did not feel antibiotics were warranted in the three clinical vignettes, 24% felt antibiotics were warranted in the ASB vignette. Suspected UTIs account for over eight million office visits and one million emergency department visits, making them among the most common infections among older adults, with an estimated annual treatment cost exceeding $2.3 billion [34]. Because ASB accounts for a sizeable portion of those prescriptions [9, 10], increasing knowledge about and changing attitudes toward ASB may represent a particularly fruitful target for future efforts.

Our study is limited by its measures, particularly the attitude questions and the use of vignettes, as well as the select population and small size. While the attitude questions are taken from prior literature [22], they do not include a scenario in which participants should want antibiotics; and while vignettes are commonly used in studies of patient and nursing behaviors [28, 35], our vignettes were not validated against actual behavior. Additionally, our sample was drawn from two community NHs and one clinic; as such, the generalizability of study results to other populations and samples is uncertain. Lastly, we did not include other populations that may influence antibiotic decisions, such as patients’ families, other nursing home patients, or physicians. While these issues limit our ability to explain antibiotic use fully, the study addresses a gap in the research evidence and provides both reassurance about attitudes to antibiotic use with older people and potential targets for future intervention.

## Conclusion

Comparison of our data with earlier reports cited above suggests that knowledge, attitudes, and behaviors towards antibiotic use in NH nurses and community-dwelling older adults have improved. While a sizeable minority of both samples expressed no knowledge of the dangers of antibiotic use, non-evidence-based attitudes towards antibiotics, and behaviors indicating inappropriate use of antibiotics, most participants’ responses were consistent with good antibiotic stewardship. However, further work on this issue is needed, given the pressing public health concern about the continued overuse of antibiotics. Thus, it is important to better understand the knowledge and attitudes of all persons involved in care decisions, to guide future antibiotic stewardship efforts.

## Abbreviations

ASB: Asymptomatic bacteriuria
NH: Nursing home
U.S.: United States
URI: Upper respiratory infection
UTI: Urinary tract infection
